# Non-Heating Alternating Magnetic Field Nanomechanical Stimulation of Biomolecule Structures via Magnetic Nanoparticles as the Basis for Future Low-Toxic Biomedical Applications

**DOI:** 10.3390/nano11092255

**Published:** 2021-08-31

**Authors:** Yuri I. Golovin, Dmitry Yu. Golovin, Ksenia Yu. Vlasova, Maxim M. Veselov, Azizbek D. Usvaliev, Alexander V. Kabanov, Natalia L. Klyachko

**Affiliations:** 1 Institute “Nanotechnology and Nanomaterials”, G.R. Derzhavin Tambov State University, 392000 Tambov, Russia; Yugolovin@yandex.ru (Y.I.G.); tarlin@yandex.ru (D.Y.G.); 2 Department of Chemical Enzymology, School of Chemistry, Lomonosov Moscow State University, 119991 Moscow, Russia; vlasova_k.y@mail.ru (K.Y.V.); veselov.mac@gmail.com (M.M.V.); azximik@gmail.com (A.D.U.); kabanov@enzyme.chem.msu.ru (A.V.K.); 3 Eshelman School of Pharmacy, University of North Carolina at Chapel Hill, Chapel Hill, NC 27599, USA

**Keywords:** magnetic nanoparticles, non-heating low frequency magnetic field, nano-magneto-mechanical activation, controlled release, apoptosis, impact localization, toxicity

## Abstract

The review discusses the theoretical, experimental and toxicological aspects of the prospective biomedical application of functionalized magnetic nanoparticles (MNPs) activated by a low frequency non-heating alternating magnetic field (AMF). In this approach, known as nano-magnetomechanical activation (NMMA), the MNPs are used as mediators that localize and apply force to such target biomolecular structures as enzyme molecules, transport vesicles, cell organelles, etc., without significant heating. It is shown that NMMA can become a biophysical platform for a family of therapy methods including the addressed delivery and controlled release of therapeutic agents from transport nanomodules, as well as selective molecular nanoscale localized drugless nanomechanical impacts. It is characterized by low system biochemical and electromagnetic toxicity. A technique of 3D scanning of the NMMA region with the size of several mm to several cm over object internals has been described.

## 1. Introduction

All conservative therapy methods can be grouped into three types according to the main approach used in them—chemical, biological/biochemical and physical [1]. Chemical methods are quite effective in many cases, but they are usually the most toxic and prone to inducing significant side effects. Biological and biochemical methods are more selective and are usually less toxic. The least toxic and safest methods are based on physiotherapy using magnetic fields (MF), but they are usually less effective, have insufficient physical background, and lack selectivity and locality.

Bionanotechnology opens new approaches that allow drastic increases in selectivity and simultaneous increases in the effects of localization up to the nanoscale and molecular levels [2,3,4,5,6,7], which also reduce the risk of organism intoxication. One of the advanced strategies is based upon functionalized magnetic nanoparticles (MNPs) that are controlled by an external alternating magnetic field (AMF) [8,9,10,11,12,13,14,15,16,17,18,19,20].

MNPs are already used to increase contrast in magnetic resonance imaging and in addressed drug delivery, including controlled drug release from the transport of nanoscale modules. Magnetic hyperthermia (MHT), which is a drugless therapy method that utilizes MNPs heated by AMF in the 100–800 kHz range, has already been developed for more than half a century [21,22,23,24,25,26,27,28,29,30,31,32]. The MNPs’ introduction to a living organism shifts the critical frequency by dividing the heating and non-heating AMF from the megahertz range to the kilohertz range. There are various combinations of MHT with thermally induced drug release from the transport of nanoscale modules [33,34,35,36,37,38,39,40].

It was reported in a number of papers that the stimulation of biomolecular systems through MHT produces more significant effects than the heating of the sample to the same macroscopic temperature in a water bath. For instance, in [38], the release rate of doxorubicin from micellar containers of 70 nm diameter, filled with magnetite MNPs coated with a hydrophobic oleylamine layer of 11 nm in diameter, was reported to be three times higher during MHT in a 330 kHz AMF than during heating to the same 45 °C temperature in a water bath. This suggests the presence of an additional factor, which, in our opinion, is related to nanomechanical magnetic activation (NMMA).

NMMA represents the other category of techniques that employ the nanoscale deformation of molecular structures by means of MNPs that are activated by non-heating low-frequency (LF) (*f* < 1 kHz) AMF, and these techniques have been developed during the last two decades [41,42,43,44,45,46,47,48,49,50,51,52,53,54,55,56,57]. NMMA utilizes the sensitivity of tissue, cells, vesicles and micelles to applied forces and induced deformations [58,59,60,61,62]. The biochemical responses to the force applied to various molecular structures in living cells are the most studied, with apoptosis receiving particular attention [63,64,65]. This type of phenomena is generally referred to as mechanotransduction [66,67,68,69]. The use of mechanotransduction opens up a wide perspective in the development of new approaches and techniques in the treatment of oncological [53,70,71] and neurodegenerative diseases [72,73,74], as well as in regenerative medicine [41,58] and other biomedical fields [52,53,54,55]. “The dark side of the force” should also be mentioned. The impact of force could possibly stimulate tumor growth due to the transmission of force from more rigid malignant cells to surrounding healthy softer ones [75].

Over several decades of magnetobiological studies, a significant amount of contradictory information and erroneous conclusions about the nature of the recorded effects has been accumulated. We will briefly discuss the most important and the most frequently occurring problems in the identification of the possible mechanisms of the impact of MF on biological objects, including those containing MNPs.

There are some sources of evidence that weak MF can produce biophysical effects in living organisms, tissues and cells, and that, in some cases, this can occur even without any MNPs [76,77,78,79,80]. These effects are hard to predict because of unclear physical mechanisms of field action. Furthermore, the reported information concerning such effects is all too often controversial and incomplete insofar as it relates to experimental conditions and the construction of reproducible independent experiments. Insights regarding the general status of the scope of magnetobiology can be gained even by examining the titles of some papers published by prominent scientists: “Why magnetic and electromagnetic effects in biology are irreproducible and contradictory?”; “Are biochemical reactions affected by weak magnetic fields?” [81,82].

A separate, yet unresolved, problem is the plausibility of the impact of the Earth’s MF [83] with *B_Earth_* = 30–50 μT, and its fluctuations reaching 2–4 orders of magnitude lower intensity even during magnetic storms [84], on various components of the Earth biosphere. Summarizing the above, it can be argued that there is no evidence and there are no generally accepted judgments about the possible mechanisms—and simple plausibility—of the effect of a weak MF on biological objects. The response of biological objects following the application of AMF depends upon a large set of spatial, temporal, amplitude and frequency characteristics of MF, including the field exposition mode, which can be continuous, intermittent or pulsed, as well as MNPs’ nature and composition, frequency windows of higher and lower sensitivity, electric and magnetic properties of the object itself, its individual peculiarities, geometry and prehistory, among other characteristics. This significantly increases the complexity of the problem.

All of these specifics distinguish the impact of vector AMF from the impacts of scalar thermodynamic parameters such as temperature, pressure, concentration, and so on. Unlike the AMF, the influence of the latter on biological objects is studied much more effectively at various scale levels, and is in good agreement with relatively simple common models and mechanisms.

Meanwhile, several magnetobiological effects are known with certainty and can be reproduced reliably. The most evident and straightforward ones, considering the underlying physical mechanism, are the induction heating of soft tissues caused by radio frequency (RF) AMF (typically 5–30 MHz in physiotherapy), and neuron stimulation caused by an eddy electric field generated by AMF pulses, with an intensity of ~1 T and a duration of ~1 ms, which is used in transcranial magnetic simulation in particular.

Many physicists question the ability of steady MF or LF non-heating AMF, with an intensity 0.1–1 T, to affect cells, tissues or living organisms, since it is hard to find a clear physical basis and molecular targets for such influence. Therefore, they consider the noticeable influence of the much weaker Earth MF (*B_Earth_* = 30–50 μT) to be even more unreasonable. The main objection is the lack of energy that MF could provide for any particles in the organism. As long as magnetically ordered regions are extremely rare or even non-existent in warm-blooded organisms, MF interacts only with objects that have magnetic momentum in the order of Bohr magneton μ*_B_* = 927.4·10^−26^ J/T, such as electrons, radicals, ions, atoms, etc. In any reasonable field with *B*~1 T their magnetic energy *U_m_*~μ*_B_B* is well below thermal energy *U_T_*~*k_B_T_R_*, where *T_R_* ≈ 300 K is ambient temperature and *k_B_* = 1.380649·10^−23^ J/K is the Boltzmann constant. Magnetic fields with *U_m_* << *U_T_* are usually referred to as thermodynamically weak, which means that they cannot significantly affect the behavior of thermodynamic systems in equilibrium. This raises questions concerning the specific non-equilibrium processes in charge of the effect and appropriate targets that are susceptible to MFs that are so weak.

**Figure 1:**
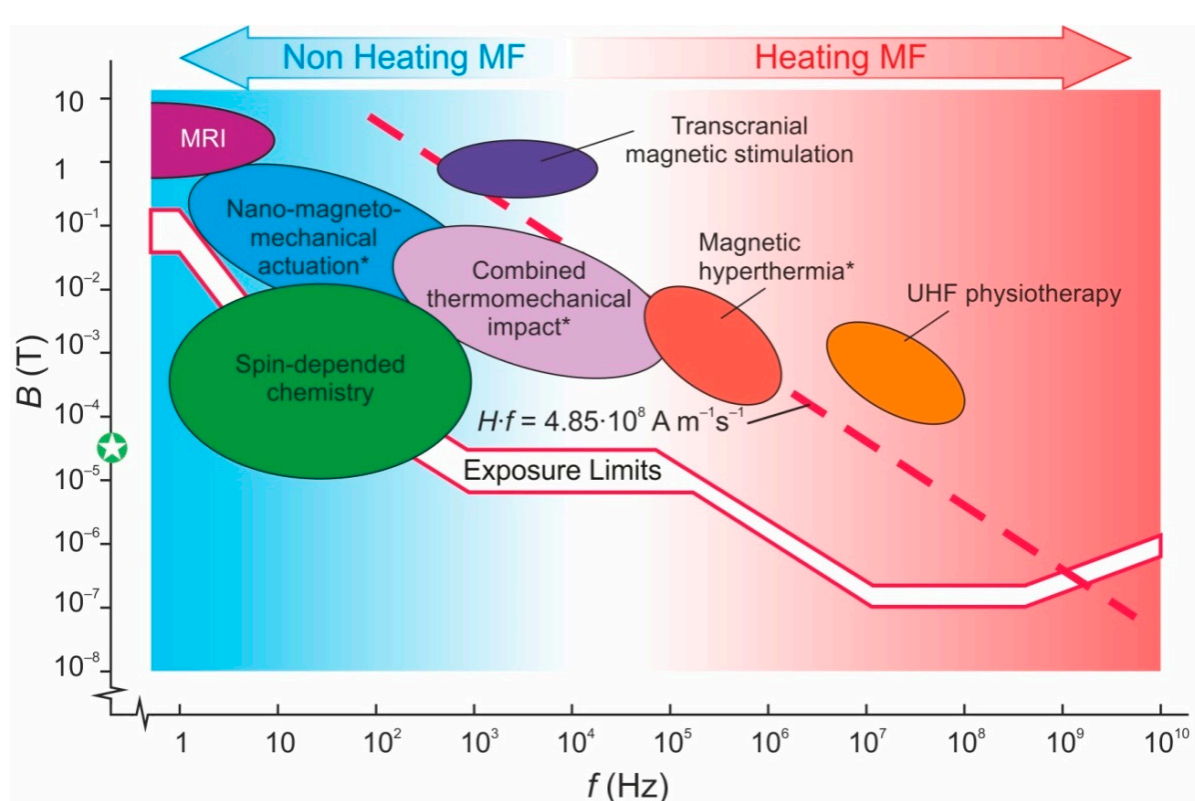
Magnetic field map with allowable intensity limits for a wide frequency range. Red broken lines indicate the maximum allowable fields according to the recommendation of the International Commission on Non-Ionizing Radiation Protection (ICNIRP) [87]: the upper line corresponds to industrial conditions and the lower line corresponds to accommodation. The dashed line corresponds to Brezovich’s condition *H*·*f* = 4.85·10^8^ Am^−1^s^−1^, which restricts the application of an AMF for medical purposes according to [90]. The green circle at the MF axis indicates the intensity of the Earth’s magnetic field. The techniques indicated by asterisks require the preliminary introduction of MNPs.

Despite the absence of commonly accepted answers to these questions, physicians, biologists, hygienists and work safety officers generally agree that hazards and risks related to the impact of AMF on the biosphere diminish with the lowering of the AMF frequency [84]. National and international guidelines and sanitary regulations [85,86,87,88] support the above relation: the lower the AMF frequency, the higher the maximal allowed field intensity both for citizens and for work staff who are maintaining electromagnetic equipment (Figure 1). Failure to understand the mechanisms of magnetic sensitivity leads to a broken dependence of the maximum permissible MF intensity on its frequency. There is no reasonable substantiation of the breaks of the curve. Let us note that several certified medical technologies significantly exceed the limit, albeit for a short periods of time (Figure 1). The AMF, when used in some medical technologies, particularly Magnetic Resonance Imaging (MRI) [89], exceeds even the empirical Brezovich threshold *H*·*f* = 4.85·10^8^ Am^−1^s^−1^, defined as a point where a human starts to feel discomfort when the AMF is switched on [90]. Here, *H = B*/μ_0_ is the magnetic field strength and μ_0_ = 4π·10^−7^ H/m is the vacuum permeability. Both International Commission on Non-Ionizing Radiation Protection (ICNIRP) limits and the Brezovich threshold take into account damage from magnetically induced electric fields, but not the hazards from direct exposure to magnetic fields, since the latter are not sufficiently justified.

Meanwhile, it is an established fact that a reduction in MF intensity significantly below the Earth’s MF, known as a hypomagnetic condition, can, in many cases, result in verifiable changes in the functioning of biomolecular structures [91].

The authors of several comprehensive papers propose a number of mechanisms of weak MF that affect biological processes, which include the formation of short living radicals and radical reactions that take place far from thermodynamic equilibrium [92,93,94,95,96,97,98,99].

These and other related papers show that the kinetics and the yield of fast radical reactions can be affected by MF even if radical magnetic energy *U_m_* << *U_T_*. The mechanisms of MF that affect processes that encompass non-equilibrium paramagnetic centers were substantiated theoretically [92,93,94]. A short explanation is as follows: the spin subsystem in dynamic processes may have insufficient time for thermalization; therefore, it can be considered as isolated from the atomic-molecular one for a period of time that is determined by the relaxation time τ*_T_*. A weak MF could affect the spinning of paramagnetic particles during that time span, so that short living radical pairs can go from singlet to triplet state, thereby preventing its recombination. However, the kinetic restraints are quite strict for such spin-dependent reactions. The lifetime of such τ*_L_* pairs should be higher than spin conversion time τ*_C_* but lower than τ*_T_*. It is unknown whether τ*_C_* < τ*_L_* < τ*_T_* conditions could be satisfied for biochemical reactions in living objects; but, for simple radical reactions in model systems, this was already proven experimentally [97,98]. On the one hand, this uncertainty prevents the development of accurate models of biological processes in weak MFs and, consequently, reasonable and reliable methods of therapy; but, on the other hand, it requires monitoring of the traces of the possible impact of MFs on biological systems in each experiment, even with a sufficiently low MF intensity.

The above energy proportion can be changed drastically through the introduction of MNPs into the system. The interaction energy of MF with MNPs, with diameters ranging from several nanometers to several tens of nanometers, is thousands of times higher than with individual electrons, thus resolving the problem of *k_B_T* as long as *U_m_* >> *U_T_*. Therefore, the magnetic energy becomes thermodynamically non-negligible and the only remaining question relates to the paths of further energy transfer into the biomolecular system. The above forms a foundation for methods of therapy that use MNPs, and it enables such methods to be advantageous compared to pure magnetic therapy.

There are at least two distinctly differing approaches to the conversion of this energy into biochemical effects. The first is its dissipation into the form of thermal energy, which takes place in magnetic hyperthermia at *f* = 100–800 kHz. The second utilizes magnetic forces more directly as local forces that induce deformation in biomolecules that are tethered or merely adjacent to MNPs rotating in non-heating low-frequency (*f* < 1 kHz) AMF. This approach is referred to as nanomechanical magnetic activation (NMMA).

Despite many attempts to provide theoretical and experimental evidence for heating localization in the volume of one cell (“intracellular hyperthermia”) [100,101], or even in the vesicle membrane [102], it was shown, both theoretically [52,54,103,104,105,106,107] and experimentally [108], that for the MNPs and MFs used in real applications, the heating cannot be localized in a region smaller than a few millimeters, and the individual MNP cannot be overheated more than by 10^−6^ °C relative to the environment. The thermal energy generated inside the MNP and in the adjacent zone is very efficiently distributed over a large area. The thermal diffusivity of any biological material differs by several times, but not by more than an order of magnitude. Therefore, during a typical experiment with a duration of ~100 s, the thermal conductivity levels out the temperature gradients in an area much larger than the cell size. In other words, adiabatic heating is possible only over a period of time that is 6–8 orders of magnitude less for a cell and 10–12 orders of magnitude less for the size scale of MNPs. To obtain a noticeable effect, one should accordingly increase the energy generation rate. An increase by many orders of magnitude in the MF intensity or the rate of energy dissipation of MNPs seems absolutely implausible, especially when taking into account all the limitations imposed by work with living organisms. We believe that the local effects observed in [100,101,102] and some other similar works are due to the rotational-vibrational motions of MNPs in the AFM. In contrast to magnetic hyperthermia, NMMA acts on a region that is comparable in size to the diameter of MNP and may have molecular selectivity [52,54,55]. This forces us to focus further discussion on the features of the NMMA of biomolecular structures in the absence of their noticeable heating.

It is likely that the first application of the nanomechanical approach that used MNPs for the generation of force was implemented by future Nobel Prize winner F. Crick in 1950 to measure intracellular microviscosity [109]. In [110], the team from Lomonosov Moscow State University reported that the activity of the enzyme can be controlled by deformation of the biomolecule. Macromolecules (MM) of trypsin and chymotrypsin were immobilized on nylon fibers and other polymer matrices with covalent bonds. Mechanical deformation of the matrix with immobilized MMs led to a decrease in the enzyme activity and an increase in its thermal stability at a deformation of about 0.05 nm, normalized to one enzyme MM. Later, this approach was developed into the field of mechanochemistry, which is associated with the immobilization of catalyst molecules on various soft materials and the control of their activity through macrodeformation of the material [56,111,112].

The first part of this mini review looks at the physical background, the second describes recent field results, and the third discusses the toxicity and other risks associated with this NMMA approach.

## 2. Theoretical Considerations

This paper does not aim at a detailed discussion of various mechanisms of energy transfer from MNPs to target objects—these can be found in papers [46,47,48] and reviews [52,54,55]. Let us mention the most important theoretical aspects discussed here.

**Figure 2:**
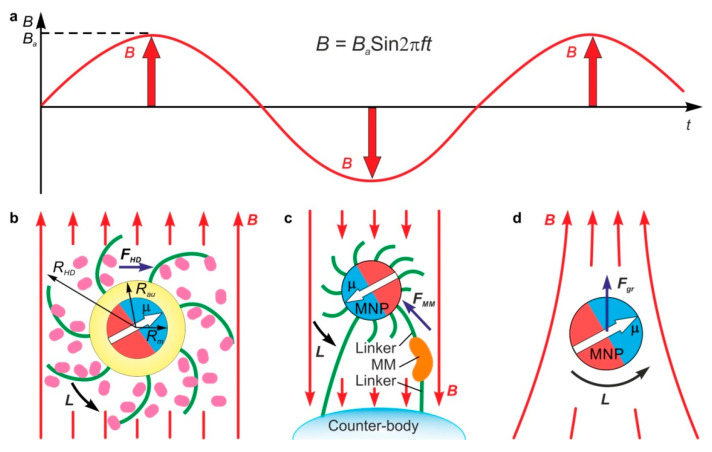
Diagram of harmonic AMF (**a**), free (**b**) and immobilized functionalized MNP (**c**) with magnetic moment **μ** in a uniform AMF ***B***, and free MNP in non-uniform AMF (**d**). *R_m_*, *R_Au_* and *R_HD_* are MNP magnetic core, gold shell ad hydrodynamic radii accordingly. ***L***—is the torque resulted from MNP-AMF interaction. The torque ***L*** produces hydrodynamic force *F_HD_* in (**b**) and contact force ***F_MM_*** in (**c**) applied to the macromolecule (MM) linked to MNP. Additional force ***F_gr_*** is applied to MNP in non-uniform AMF (**d**).

In the general case, to any body/particle with magnetic moment **μ** exposed to a magnetic field ***B***, a torque ***L* = μ*xB*** and a force ***F_gr_* = (μ∇)*B*** are exerted (Figure 2). In a uniform MF, only a torque is applied to the body because all spatial derivatives of the field are equal to zero. In contrast to solid-state magnetic elements, MNPs located in a suspension or a tissue are surrounded by a liquid or viscoelastic media; therefore, under the influence of AMF, they can vibrate in different modes. The nature and amplitude of a free MNP motion in AMF depend on its hydrodynamic radius and magnetic moment, the viscoelastic properties of the environment, the MF intensity and frequency, the initial angle between vectors **μ** and ***B***, and other factors.

It should be noted that the above equations for ***L*** and ***F*** correspond to the maximum induced values if **μ** is assumed constant. Actual values can be significantly lower due to magnetic relaxation, i.e., the rotation of the magnetic moment vector of the MNP inside MNP body as a result of its interaction with MF. The details of this process are determined by many factors, including the radius of the MNP core and the magnetic properties of the material, AMF frequency, media viscosity, and others.

While the specifics of magnetic relaxation are not very important for heating the MNP in AMF, and energy dissipation is roughly proportional to the AMF frequency, for NMMA, the difference between Neel and Brawn relaxation is crucial because Neel relaxation leads to a rapid rotation of the vector **μ** without rotating the MNP itself. It reduces the torque and other external manifestations of MNP–MF interaction. Some torque still also exerts MNP in this case, but it is the result of a residual vector alignment mismatch, while the large theoretical value applies only for a short part of the period, which is proportional to the ratio of the Neel relaxation time to the AMF period. The most important parameter separating the Neel and Brawn relaxation regions is the MNP magnetic core radius *R_m_*, which must be higher than a certain critical value, namely R_m_*, to freeze out the Neel relaxation. R_m_* depends on the magnetic core material, the hydrodynamic radius of MNP, and the viscosity of the environment. The most common material in biological applications is magnetite, and, for a typical MNP geometry and environment, R_m_* ≈ 6.5 nm.

The other important aspect of the interaction of MNP with surrounding molecules is the mechanical constraints imposed on the movement of MNP. There are two typical cases. One is the case of free MNPs suspended in the liquid (Figure 2b), and the other is that of MNPs anchored to other bodies, including other MNPs, vesicles, cell membranes, microfibers, etc. (Figure 2c). In addition to torque ***L*** and ***F_MM_***, the force ***F_gr_*** is arisen in non-uniform AMF (Figure 2d). It oscillates in phase with the AMF (Figure 2a) and causes MNP reciprocal motion.

Free MNP cannot produce torque that is higher than the torque exerted by its viscous friction during rotation. Force evaluation gives values of *F_HD_*—the hydrodynamic force applied to MM—of no more than 1 pN for realistic experimental conditions, but this value can sometimes be sufficient for the acceleration of the gradual washout of the therapeutic agent, from the polymer shell of the MNP, in order to implement controlled drug release [113]. In the case of anchored MNP, the mechanical properties of the molecular tether, the location of its joint points on the MNP and counterbody, and other link parameters, become important. A detailed description of the dynamics of free and anchored MNPs in AMF can be found in [48,52,53,54,55,114].

There are other important concerns when considering the optimal choice of AMF frequency for NMMA. Inertial and hydrodynamic forces grow with the increasing of *f*. The inertial force for MNP with a 10 nm radius is much less than the magnetic and hydrodynamic forces at AFM frequencies of up to many megahertz; therefore, they can be neglected in the implementation of NMMA.

**Figure 3:**
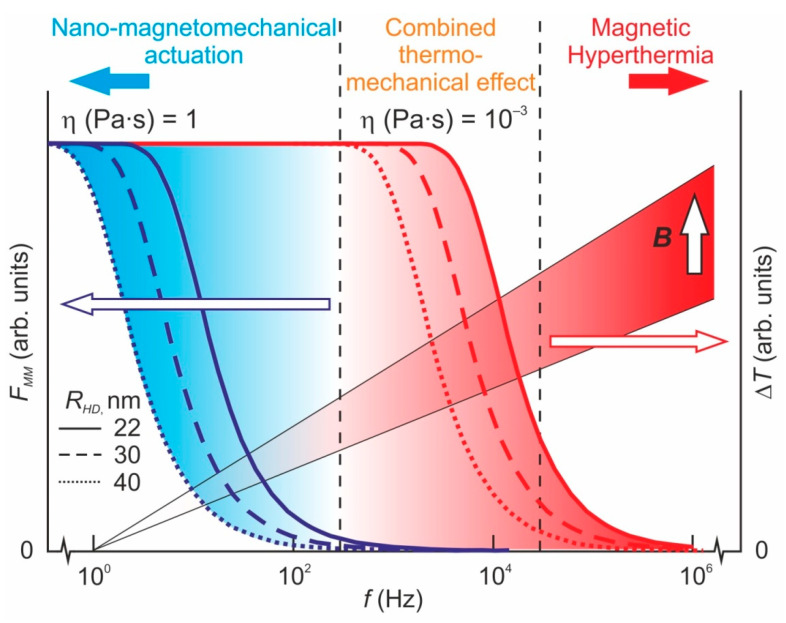
Frequency dependence of the heating Δ*T* and the range of angular oscillations of MNPs with the magnetic radius of *R_m_* = 10 nm and various hydrodynamic radii *R_HD_* in the media, with various microviscosity values η, under the action of harmonic AMF *B* = *B_a_*Sin2π*ft*; *B_a_* = 100 mT.

To reduce energy loss due to heating and dissipation processes, the AMF frequency *f* should meet the condition *f* < *f_c_*, where *f_c_* = μ*Bk*(12πη*V_HD_*)^−1^ is some characteristic frequency determined by MF intensity *B*, medium microviscosity η, MNP form factor *k*~1 and its hydrodynamic volume *V_HD_* [46,47,48,52,54]. At this frequency, the maximum torque resulting from the MF–MNP interaction becomes equal to the maximum possible torque of the viscous forces that are applied to the rotating MNP by surrounding media. Thus, the larger the hydrodynamic radius of the MNP, the lower the AMF frequency that should be chosen. Anchored MNP can exert a maximum contact force *F_MM_* of up to F*_MM_ = μ*B/R_HD_* on the binding macromolecule, and this can even be slightly higher with a special binding geometry. If the AMF frequency approaches or exceeds *f_c_*, the *F_MM_* force diminishes due to viscous friction forces. The behavior of rod-shaped MNPs in AMF is even more complicated, which opens additional possibilities for controlling the effect of AMF. There is some evidence that such MNPs may be more effective than spheroidal ones [115,116,117,118], whereas rod-like MNPs do not require strong bonds to MM or other particles to induce significant deformation. Thus, the MNP and AMF parameter requirements for MHT and HMMA applications are exactly the opposite (Figure 3).

The next stage in the development of this technology is the choice of MNP and AMF parameters in order to produce the desired NMMA response. There is a large amount of experimental data on changes to the properties of individual macromolecules, molecular structures and living cell responses to an application of force [36,49,60,119,120,121,122,123]. Usually, such information is obtained using single molecule force spectroscopy (SMFS), implemented by optical or magnetic tweezers, or atomic force microscopy in contact mode. It should be mentioned that it is impossible to determine precise values of critical force *F_c_* that are sufficient to cause any significant effect because molecular effects are of a statistical nature and are characterized by large dispersions even in completely identical experimental conditions. Furthermore, for any given process, the *F_c_* value depends upon a large variety of parameters including the load frequency, the application rate and duration, and the previous history, as well as such environmental parameters as temperature, pH and others. Therefore, they are usually presented as histograms or intervals of the most probable values. Typical values of *F_c_* for some molecular structures and processes are presented in Table 1. As can be seen in this table, to control all of the processes of interest for biomedicine, at the scale of individual molecules, molecular structures and cells, forces in the range between tenths and several hundreds of piconewtons are needed, which are easily achievable with reasonable MNPs and AMF. It follows from Table 1 that the dangerous cleavage of covalent bonds requires forces of several thousands of piconewtons. This value is hardly reachable in NMMA with the currently used MNPs and AMF.

## 3. Some Experimental Results

In the physics of nanostructures, microelectronics and photonics, the approach that uses controlled elastic deformation to manipulate material properties was, in recent years, referred to as straintronics [124]. Though it is fundamentally important and used in real applications, straintronics in the form described in [124] is hardly applicable to biomedical problems as long as substrate deformation requires macroscopic loading units that are incompatible with living organisms. The employment of MF-activated MNPs as a nanoscale deformation machine allows the implementation of various specific loading schemes for any biomolecular structure, from individual bioactive MMs to cells [52,53,54,55,56,57]. They can, through the use of mills, attritors, etc., provide nanoscale locality and a degree of selectivity that is unreachable for traditional mechanochemistry, and can, unlike SMFS techniques that deal with isolated single MM, also provide mass procession as a result of the large number of MNPs in suspension.

**Figure 4:**
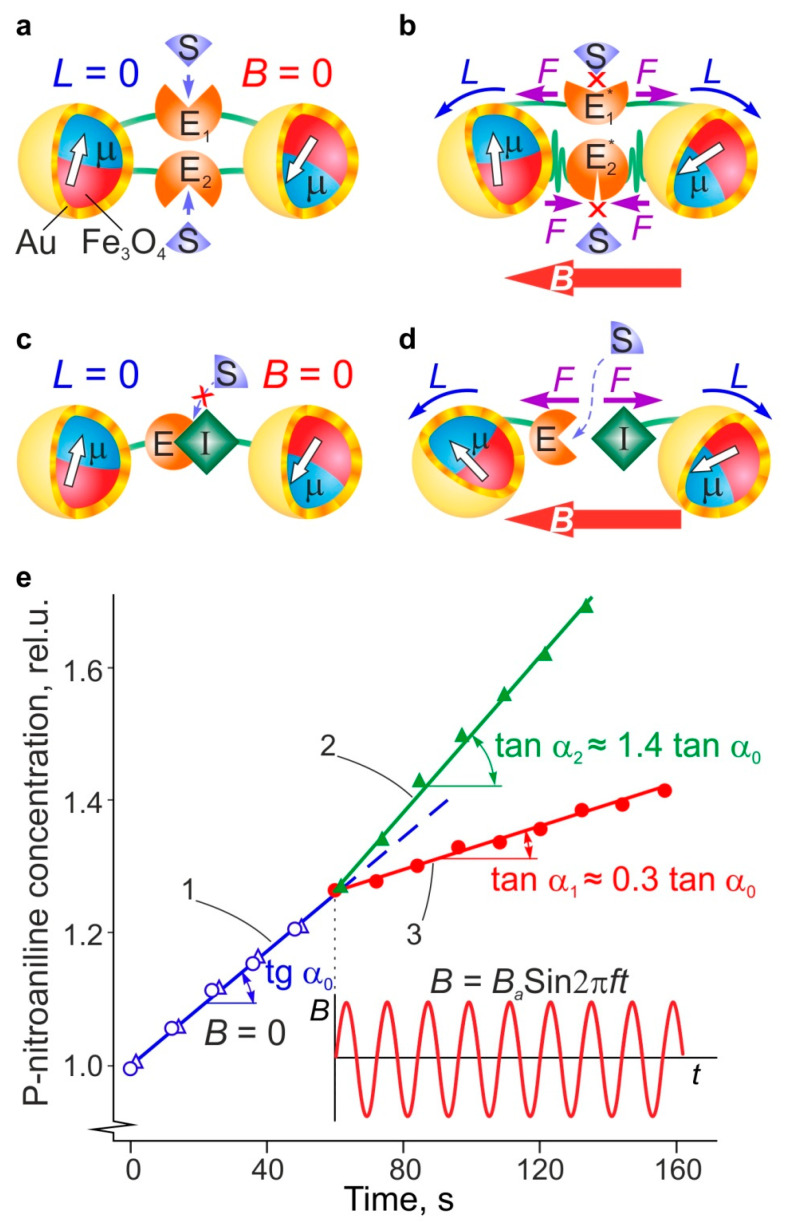
Effect of a LF MF (*B* = 88 mT, *f* = 60 Hz) on the catalytic activity of chymotrypsin macromolecules immobilized in the dimer complex of two MNP: (**a**) diagram of dimer complex without MFs and (**b**) upon exposure to a MF; (**c**) diagram of the f-MNP-ChT-TI-MNP complex without a MF and (**d**) upon exposure to a MF (E is enzyme, S is substrate, I is inhibitor, TI is Trypsin inhibitor, *L* is the torque applied to MNPs in LF MF, and *F* are forces acting on macromolecules in LF MF); (**e**) the kinetics of the light absorption growth by the product formation during the biocatalytic reaction before the switching on of LF MF (the dependence (1)) and during exposure to the field (dependences (2) and (3) referring to the complexes in (**d**) and (**b**), respectively).

In one series of experiments described in [56], the mechanochemical effect of MF on the catalytic activity of chymotrypsin (ChT), which was immobilized on magnetite nanoparticles and coated with gold and polyethylene glycol (PEG) ligands of 2–5 nm length, was studied. Some part of the ChT MMs formed bridges that connected two MNPs, thereby producing MNP dimers (Figure 4). The catalytic activity of immobilized ChT and its change during exposure to MF were recorded using a spectrophotometer that measured the rate of colored p-nitroaniline product formation during the catalytic hydrolysis of the N-succinyl-L-alanyl-L-alanyl-L-prolyl-L-phenylalanine (SAAPFpNA) substrate. The reaction rate, which was proportional to the slope of the kinetic curve, was reduced threefold with the application of MF (Figure 4e, dependence 3). Both the heating of MNP and the suspension volume in MF, with such low frequencies and intensities as those used in the experiment, were below 0.1 K, as measured directly using the remote infrared temperature gauge, and could, therefore, be neglected. Thus, the effect, due to its sensitivity to the ChT concentration and LF AMF parameters, could only be ascribed to the mechanical impact of the rotational oscillations of MNP, which were induced by AMF, on the conformation and active centers of ChT MMs.

In the other set of experiments described in [56], ChT and trypsin inhibitor (TI) molecules were immobilized on two separate MNP ensembles. Their mixing resulted in the formation of a ChT-TI complex with low activity within the MNP-ChT-TI-MNP dimer aggregate. To measure the activity of the ChT-TI complex, a SAAPFpNA hydrolysis reaction and spectrophotometric monitoring of the formation of p-nitroanaline products were used as described above. Enzyme activity increased during exposure to *B* = 88 mT, *f* = 60 Hz MF, by a factor of 1.4 (Figure 4e, dependence 2). The absence of a noticeable increase in temperature makes it reasonable to ascribe the increase in activity to the cleavage of the ChT-TI bond in the MNP-ChT-TI-MNP complex and the unblocking of the enzyme active center. It should be noted that the dependence of enzyme activity on MF intensity reaches saturation at some point, depending on the character of the activated process.

A number of papers described the magnetomechanical activation of MNP through the application of LF AMF with the empirical choice of experimental parameters [43,44,45,46,47,48,49,50,51]. Specifically, the authors of [51] mentioned the use of NMMA in regenerative medicine and tissue engineering, and the controlled release of DNA and other bioactive molecules from nanocontainers was pointed out in [44,125]. Changes in enzyme activity were reported in [45,56]. A number of publications [43,53,126,127] reported the apoptosis or necrosis of malignant cells induced by NMMA. The MF intensity and frequency dependences of this effect were found to be complex and even non-monotonic, so thorough systematic studies of the effects of the reactions of various biochemical systems on NMMA are necessary for the future application of frequency-selective nanomechanical impacts on molecular structures that are localized at the nanoscale. It should be noted that this is principally impossible for MHT. Taking the above into account, and employing the described models, more physically substantiated experiments were carried out [53,55,56,117,128]. The conditions for the most effective NMMA application found in these papers were generally in agreement with our theoretical considerations, which were presented in [46,47,48,115,129,130,131]. For example, the MF-induced release—and change in the activity—of SOD1 enzyme MMs from MNPs coated with poly(lysine)-poly(ethylene) glycol block copolymer carriers were studied in vitro, and were found to depend on MF parameters in the intensity range of 8–125 kA/m and the frequency range of 30–410 Hz in [128]. It was shown that SOD1 desorption depends upon the duration, intensity and frequency of MF exposure and results in an increase in enzyme activity. For instance, after suspension exposure in MF with *H* = 55 kA/m and *f* = 50 Hz for 30 s, SOD1 activity increased by 15% due to enzyme desorption. The effect was found to be reversible, and after the switching off of MF, SOD1 begun to sorb back onto the corona of the MNPs polymer due to electrostatic interaction, and its activity was thus reduced.

Another experiment demonstrated the applicability of MNP, activated by non-heating AMF at a frequency of 50–400 Hz, to the controlled release of the desired agent from liposomes, by means of the alteration of the microviscosity of the lipid membrane. Liposome-MNP complexes were formed due to the electrostatic interaction between dopamine bound to MNPs and lipid anionic units (mostly phosphates). A 5 min exposure to AMF at a frequency of 50–150 Hz resulted in the release of the cargo of low molecular weight compounds, as reported in [117], for sodium chloride. The effects depended upon the frequency and intensity of AMF (see Table 2). As shown by infrared (IR) spectroscopy, the oscillation of MNP in AMF results in the loosening of the liposome membrane, which resembles phase transition [117].

NMMA has the potential to modulate the functioning of nerve tissues [73]. For example, MNPs activated by ~50 Hz AMF were employed to stimulate ionic channels and stem cell differentiation. This is of great interest in terms of the non-invasive treatment of various neurodegenerative diseases [132,133,134,135].

The other prospective application of MNPs in biomedicine is neuron regeneration and growth engineering [73,136,137]. As shown in [136], the introduction of MNPs into neurons allows the control of the direction of axon growth by means of gradient MF. A similar effect, accompanied by the acceleration of cell differentiation as a result of growth hormone receptor stimulation, was reported in [137]. There are other approaches to neural modulation by means of the mediation of AMF by MNPs [74].

The NMMA approach has many advantages compared to such widespread methods as chemotherapy, radiotherapy, photothermal therapy, photodynamic therapy, and ultrasound, as well as other methods that use MNPs and AMF, as summarized in Table 2.

The main advantages of NMMA over technologies that use heating radiofrequency AMF with the frequency above hundreds or thousands of kHz, such as MRI or MHT, include its higher flexibility and generality, its ability to reach molecular level locality and selectivity, as well as its use of safer non-heating LF AMF. In addition to use by itself, NMMA can fit in with traditional therapy (addressed drug/gene delivery, controlled drug release, changing of cell behavior [19,41,42,43,44,45,49,53,56,73,117,128]) easily. Furthermore, LF MF is much safer than heating radiofrequency MF; thus, sanitary regulations and norms, and international regulations and recommendations, allow much higher intensities of the former in the environment. The primary mechanism of the non-heating effect of LF AMF is the mechanical activation of individual MMs, transmembrane proteins, ionic channels, and cell membrane receptors. This requires an intelligent choice of MF parameters and specific MNP functionalization, thus allowing the deformation of conjugated and adjacent bioactive MMs, and thereby changing its conformation, relative position and the functioning induced by the rotational oscillations of MNP in AMF. It is the option of linking the specific biomolecules that can target other very specific molecules in biological systems to MNP that makes it possible to reach very accurate targeting of the effect of AMF in NMMA at the nanoscale level, unlike that of MHT, which cannot be localized at better than ~1 cm^3^ volume due to the high thermal conductivity of the tissue, and has no biochemical selectivity. As shown experimentally, due to the high sensitivity of biological molecules to applied force and strain, NMMA can change enzyme activity, and loosen vesicle and cell membranes, thereby increasing their permeability, as well as affecting cell metabolism, fighting with malignant cells and ignoring healthy ones. Moreover, all these effects do not require any heating, ionizing radiation or very toxic therapeutic agents, the impact of which cannot be localized at a molecular or cell level, which leads to various dangerous side effects.

There are just a few papers reporting studies NMMA in vivo (see the review [50]), while in vitro studies are more numerous. Experiments on cells and cell cultures showed the effectiveness of NMMA as a means of inducing apoptosis in cancer cells [36,37,41,42]. In addition, there is indirect evidence of the possible participation of drugs containing iron ions, or natural iron-containing proteins-ferritins, in the mechanisms of MF action in vivo [138,139,140,141]. Of course, for a reliable proof of the magnetomechanical origin of the effects observed in vivo, it is necessary to conduct targeted experiments with the introduction of optimally designed MNPs, with known magnetic characteristics and functional shells, into the body, as well as physically substantiated parameters of the activating AMF. This will bring greater certainty to the situation and will help both to better understand the mechanisms of action of AMF and the means of increasing its effectiveness in vivo.

## 4. Toxicological Aspects

There are three subjects in magnetic therapy that use MNPs as a mediators and concentrators of AMF action: MNPs, AMF and the results of their interaction. As for MNPs, they have, by now, been widely used in MRI diagnostics, iron-deficient anemia treatment, addressed drug delivery, cell sorting and other biomedical applications for a long time [8,9,10,11,12,13,14,15,18,19,20].

A number of studies claimed that MNPs are nontoxic or even useful [142,143,144]. However, there are even more papers declaring some indication of MNPs’ cytotoxicity in vitro and in vivo [145,146,147,148,149,150]. There are several Food and Drug Administration and European Medicines Agency (FDA/EMA)-approved iron oxide nanomaterials (FerrlecitVR, VenoferVR, INFedVR, DexferrumVR, FerahemeVR, Feridex I.VVR, ResovitVR, GastromarkTM, and Ferumoxtran-10) that are employed to treat iron deficiency in chronic kidney disease, imaging of liver lesions, and lymph node metastasis imaging [151]. Some of them, however, were discontinued because of side effects and toxic effects shown in vivo.

A number of reviews, in recent years, were devoted to the analysis of various factors affecting the toxicity of magnetic nanoparticles [10,152,153,154,155,156,157].

These reviews summarize the information on the toxicity of MNPs. There are no doubts that some kinds of nanoparticles have demonstrated such toxic effects as inflammation, ulceration, decreases in growth rate, declines in viability and triggering of neurobehavioral alterations in model animals (see [152]). The toxic effects of MNPs in vivo were discussed in detail in several reviews [145,152,154,156]. Organ-specific toxic effects and routes of introduction were discussed. Magnetite MNPs coated with polymers containing polyethylene oxide showed no toxicity on different organs in mice even after 14 days [154].

It is, therefore, important to understand the toxicity of MNPs, as it depends on such factors as size, charge, shape, structure, surface modification, concentration, dosage, biodistribution, bio-availability, solubility, immunogenicity and pharmacokinetics. As shown in many reports, iron oxide-based materials such as magnetite and maghemite are considered safe and are also currently in clinical use as MRI contrast agents [158].

Magnetite (Fe_3_O_4_) and maghemite (γ-Fe_2_O_3_) are considered the most suitable materials for the synthesis of MNPs. However, uncoated MNPs are quite toxic, as shown in many studies [152,153]. To ensure stability, as well as to increase bioavailability, various coatings are used: natural (carbohydrates, proteins) and synthetic polymers (polyethylene glycol (PEG), polyvinyl alcohol (PVA), polyvinyl pyrrolidone (PVP), copolymer of lactic and glycolic acid (PLGA), and noble metals (gold and silver) (see examples in recent papers [56,159] and reviews [10,152]).

Gold nanomaterials, assembled with magnetic iron oxides cores to provide gold–iron oxide hybrid structure MNPs, offer benefits, including the easy chemical modification of gold surfaces, which are suitable for drug delivery, spin dynamics and plasmonic applications.

PLGA and Polylactic acid (PLA) polymers are FDA-approved based on the simplicity of their particle formulation and non-toxic biodegradation products. These polymers are used to coat the unstable reactive surface area of MNPs to stabilize them for such in vivo functions as drug delivery or gene delivery by adsorbing proteins or loading drugs [160]. The magnetic cores of these fabricated MNPs help in their accumulation at a desired site and the unloading of the drug molecules at this site is controlled by an external magnetic field [10,161,162].

Surface coating with PEG was shown to reduce the interaction of MNPs with plasma proteins, decreasing the chances of internalization and clearance by macrophages [163,164]. γ-Fe_2_O_3_ nanoparticles coated with polymers containing polyethylene oxide (5 and 15 kDa) were not toxic towards prostate cancer cell lines, human umbilical vein endothelial cells (HUVECs), and human retinal pigment epithelial cells (HRPEs), even after the uptake of MNP into such cells [165].

As was found for long exposure times (48 h), the cytotoxicity of iron oxide nanoparticles can be ascribed to free radical production, but this toxic effect may be neutralized through the use of polyethylene glycol modification [155].

Albumin nanoparticles’ coating was also shown to give a stable and biocompatible shell that prevents cytotoxicity of the magnetite core [155]. The authors synthesized bovine serum albumin-coated iron oxide nanoparticles with two different sizes, 80 and 40 nm, and a polyethylene glycol derivative of the latter one. A number of in vitro toxicological tests on human fibroblasts and U251 glioblastoma cells were performed. A simple survival assay of both the cells and the lactate dehydrogenase (LDH) activity after 24 h of incubation showed no significant loss in the confluency area of the human fibroblast (HF) and human glioblastoma U251 cells at all MNP concentrations. However, at 48 h, the highest concentration of BSA-MNP-80 and BSA-MNP-40 showed some cytotoxic effect, which was stronger in the case of BSA-MNP-40 in HF cells (no toxicity shown in U251 cells).

Superparamagnetism is an important feature in terms of avoiding agglomeration and directing MNPs to site-specific locations inside the body. Superparamagnetism arises from the magnetic material core of the MNPs and depends on the core size. As shown above, MNPs of different sizes revealed different toxic effects [155]. Other studies confirmed that the particle size may, indeed, have different effects on cells in vitro. For instance, 30-nm-sized MNPs showed relatively higher toxic effects as compared to those of 0.5-μm-sized particles. When incubated with the A549 alveolar epithelial cell line, a size-dependent and dose-dependent influence on cell damage was observed [10]. For instance, 30-nm-sized Fe_3_O_4_ particles caused higher oxidative DNA damage compared to 0.5-μm-sized particles at 80 μg/mL concentration, while, at lower concentrations such as 40 μg/mL, none of these particles were toxic [10]. Additionally, at 80 μg/mL concentration, both 30-nm- and 0.5-μm-sized Fe_3_O_4_ particles showed mitochondrial depolarization, suggesting mitochondrial damage with subsequent cell death.

The adsorption of plasma proteins (opsonins) onto the NPs surface (resulting in their recognition by macrophages and systemic clearance) depends on the size of the particles. For instance, it was shown that the quantity of plasma protein adsorbed was lower for the smaller NPs (6% of protein adsorbed onto 80-nm-sized particles), whereas it was significant for the NPs of relatively larger size (23% and 34% of protein adsorbed onto 171-nm- and 240-nm-sized particles) [10].

Summing up the above, one can assume that the most widely used MNPs, consisting of a magnetite core, gold coating and polymer shell, are characterized by low toxicity both in vitro and in vivo. Some of them are approved by the FDA, which allows their use as a suitable instrument for the implementation of biomedical technology platforms based on NMMA. Unlike MHT with steadily growing temperature, NMMA methods that use non-heating AMF act by means of forces that are localized at comparable distances to the MNP radius and that are almost independent of exposition time and MNP concentration. Furthermore, NMMA requires a significantly lower MNP concentration than MHT. Altogether, it greatly reduces the risk of overdose.

Risks of exposure to activating AMF by itself are assessed according to sanitary norms and regulations. As mentioned above, the lower the AMF frequency, the lower the risks. Mechanical activation employs fairly safe, very-low-frequency MF (usually 1–100 Hz) with an intensity in the range of tens to hundreds of milliteslas.

To further lower the requirements for AMF, one can use MNPs made of material with higher saturation magnetization than that of widely used magnetite [166]; however, their toxicity is studied to much lesser extent than that of iron oxides.

**Figure 5:**
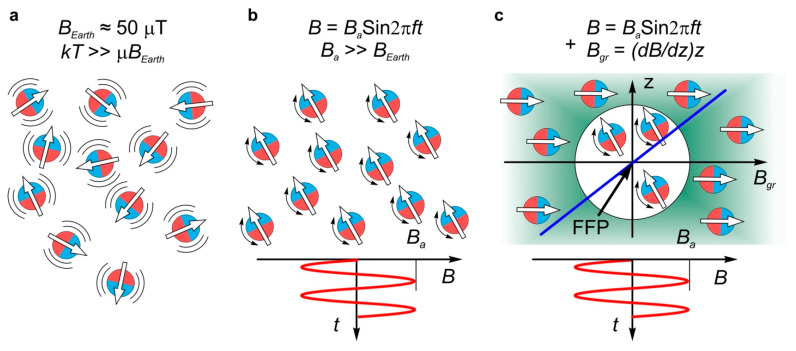
Focusing principle of the effect of AMF on MNPs employing gradient-steady MF *B_gr_* [163]. (**a**) Thermal fluctuations of MNP in the absence of external MF. The natural Earth MF *B_Earth_* is neglected; (**b**) rotational oscillations of MNP forced by activating AMF in the whole volume occupied by fields and particles; (**c**) oscillations of MNP forced by activating AMF in the presence of focused, gradient-steady MF *B_gr_* are possible only in the region where *B < B_gr_*. Blue line shows the dependence of *B_gr_* on distance from FFP.

A significant further reduction in the body burden imposed by NMMA treatment can be achieved by employing an additional gradient, MF *B_gr_*, with a field free point (FFP) [167]. The FFP can be localized anywhere within the object under AMF treatment (Figure 5). Such a field can be generated by two or three pairs of Maxwell coils, which are Helmholtz coils with opposite current directions, for FFP repositioning. Mechanoactivation in such fields can take place only near the MF FFP gradient, where the *B_gr_* intensity is lower than that of the activating AMF. By adjusting the currents in the coils, one can control the size of the processed area (typically from several mm to several cm), as well as its position. This allows 3D scanning in the therapy process. This approach is already used for the 3D visualization of the biodistribution of MNPs in the body, with a rate of up to several tens of images per second [168,169,170,171,172]. The rate is thousands of times higher than that obtained in MRI. The same principle of focusing the impact of AMF in a given region within the object can also be employed in active therapy methods such as MHT [173] or NMMA [167].

## 5. Conclusions

The paper discusses and summarizes research on the features related to the use of MNPs as transducers of non-heating LF AMF energy into the deformation of bioactive MMs, bionanostructures and cells linked to the MNPs. The forces and deformation of these objects are numerically estimated for magnetite MNPs. It is shown that easily available LF AMF, with frequencies in the range of several to hundreds of hertz and induction in the range of several to hundreds of milliteslas, can force 10–30 nm functionalized magnetite MNPs to activate almost all important biochemical processes from the level of MMs to that of cells. This approach provides a toolbox to change the techniques related to the remote enzyme activity, addressed drug delivery, controlled release and drugless destruction of malignant cells, which can provide a new foundation and a great perspective for next generation therapies. The strategy described above allows the implementation of a large set of diagnostic, therapeutic and monitoring functions within the same technological platform, which is safer and causes less side effects than the disconnected set of existing methods. To adopt this strategy in medical practice, one should further develop both the theory and biochemical experiments, and advance from the laboratory to the clinic.

It should be noted that MF physiotherapy, the spin-dependent kinetics and yield of biochemical reactions, magnetic hyperthermia and nano-magneto-mechanical activation use quite different means of biostructure control. The terms “magnetobiology”, “magnetic impact” and “magnetic effects” only cover these differences very formally, while the underlying mechanisms of action of MF on biological objects differ greatly. The MF itself, with usual laboratory intensities of up to several tesla and much lower typical values such as 0.01–0.1 T, is a thermodynamically weak factor for biological systems. The responses to exposure to such fields are hard to reliably reproduce, and can sometimes be completely indistinguishable. However, the possibility of their reproduction makes control experiments necessary in any case. The impact of MF on biological objects that can be registered reliably is explained by the indirect effects of MF, comprising an eddy electric field and heating in the vast majority of cases. Therefore, the effects specific to magnetism are negligible or missing completely. The introduction of MNP allows the condensing of MF energy in nanoscale volumes by many orders of magnitude and enables new nanomechanical factors (forces and deformations). This factor has no magnetic specifics, but unlike eddy electric fields and heating, it can be localized at a molecular or a cell level. Nanomechanical actuation has much higher selectivity and can induce a much wider variety of effects than temperature increases due to the sensitivity of the molecular system to the force frequency, amplitude and application point, as well as the tensor character of the induced deformations. Without detailed descriptions of experimental conditions and a good understanding of the mechanisms of the impact of AMF, it is impossible to optimize anticipated responses and build any therapeutic platform using AMF as an energy source.

## Figures and Tables

**Table 1:** Typical values of magnetic-field induction necessary for the activation of some processes in living cells (in the case of MNPs with *R_m_* = 10 and 15 nm).

No	Process	Evaluation of Necessary Magnetic-Field Induction *B*, mT		Typical Experimental Threshold Force Measured by SMFS Methods, *F*, pN	Reference on Experimental Measurement of Force
		*R_m_* = 10 nm	*R_m_* = 15 nm		
1.	Activation of various ionic channels	1.35–67.5	0.6–30	0.2–10	[49,119,120]
2.	Protein–protein interaction	6.75–67.5	3–30	1–10	[60,121]
3.	Activation of membrane receptors	67.5–337.5	30–150	10–50	[36,49,119,120]
4.	Bond cleavage between transmembrane protein and membrane	202.5–337.5	90–150	30–50	[120,121]
5.	Antigen–antibody interaction	67.5–675	30–300	10–100	[60,122]
6.	Onset of unfolding of protein macromolecule	135–675	60–300	20–100	[60,123]
7.	Interaction of ligand with receptor	6750	3000	~1000	[120,122]
8.	Interaction of protein with lipid	337.5–675	150–300	50–100	[121,122]
9.	Breaking of covalent bond	6750–33,750	3000–15,000	1000–5000	[60,120]

**Table 2:** Comparison of different methods of producing magnetic effects on biochemical systems.

Method	Advantages	Drawbacks	Typical MF Parameters
Magnetic hyperthermia	Versatility, ease of implementation	Need to introduce MNPs, difficulty in controlling temperature and dosage, low locality, non-specificity, risk of damage to healthy tissues	*f* = 100–800 kHz *B* = 5–30 mT
UHF physiotherapy	Ease of implementation, noninvasiveness	Unsafe field frequencies, non-specificity	*f* = 26–40 MHz *B* < 0.1 mT
Transcranial magnetic stimulation	Noninvasiveness	Non-specificity, insufficient localization	*f* = 1–10 kHz *B* = 1–3 T
Nano-magnetomechanical actuation	Molecular locality, high specificity, safe frequencies, multimodality	Need to introduce MNPs	*f* < 1 kHz *B* = 10–500 mT
Spin-dependent chemistry	No mediators needed	Difficulty of control, ability to regulate only some reactions	*f* = 0–100 Hz *B* < 10 mT
